# Heparan Sulfate Differentially Regulates Fibroblast Binding by Extracellular Vesicles and Human Cytomegalovirus

**DOI:** 10.1002/jev2.70390

**Published:** 2026-11-03

**Authors:** Vladimir Bokun, Antonio Di Maio, Yibing Zhang, Wengang Chai, Beth Holder, Yan Liu

**Affiliations:** ^1^ Institute of Reproductive and Developmental Biology, Department of Metabolism, Digestion and Reproduction, Faculty of Medicine Imperial College London London UK

**Keywords:** cell binding, cytomegalovirus, extracellular matrix, extracellular vesicles, glycosaminoglycan, heparan sulfate, virus

## Abstract

Extracellular vesicles (EVs) are lipid‐enclosed particles produced by cells as a vehicle of intercellular communication and cargo exchange. Virus‐infected cells produce EVs with altered cargo, which can include viral components. These EVs play functional roles in multiple aspects of viral infection. Their interactions with cells, including those occurring at the cellular surface prior to internalization, which are key to their subsequent functional effects, have not been studied extensively.

In this work, we investigate the role of cellular glycosaminoglycans (GAGs)—major constituents of the extracellular matrix (ECM)—in cell binding by EVs produced by homeostatic cells and those infected with human cytomegalovirus (HCMV). We show that EVs from both HCMV‐infected and uninfected cells interact with heparin, a highly sulfated form of heparan sulfate. However, in the context of binding to fibroblast cells, we show that heparan sulfate on both the cell and the EV surface inhibits EV‐cell interactions, while promoting attachment by human cytomegalovirus. Instead of GAGs, EVs were found to interact with other ECM constituents, namely collagen and fibronectin, on the fibroblast surface.

This work shows that heparan sulfate serves as a bi‐directional negative regulator of fibroblast binding by EVs, while acting as a positive regulator of HCMV attachment. Thus, EVs and HCMV appear to utilize distinct, non‐overlapping ECM ligands for cell binding, in both homeostasis and during HCMV infection.

## Graphical abstract

**A model of EV and HCMV interactions with the fibroblast extracellular matrix**. Heparan sulfate inhibits interactions between EVs and fibroblast cells bi‐directionally, while serving as the major cell attachment target for human cytomegalovirus (HCMV). EVs instead interact with collagen and fibronectin to facilitate fibroblast binding. Created with BioRender.com.

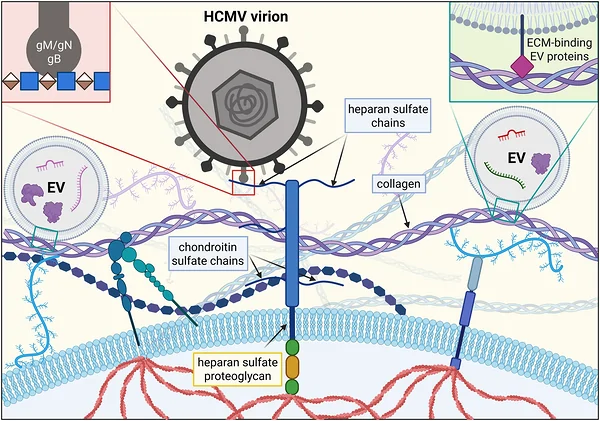

## 1 Introduction

Extracellular vesicles (EVs) are constitutively secreted by all cells and serve as a key mode of communication in both homeostatic and pathological conditions (Anand et al. 2019; Atai et al. 2013). Some of the functions of EVs are dependent on their cellular uptake, which is preceded by interactions between the EV and the cell surface (Buzás et al. 2018; Wang et al. 2025). However, EVs could also trigger cellular responses through surface interactions alone. Both cells and EVs possess complex surface proteomes, including various extracellular matrix (ECM) components, which may mediate these interactions (Buzás et al. 2018). Similarly, viral infection also requires initial interactions between viral particles and the cell surface before virus entry (Koehler et al. 2020). Since EVs and viruses are co‐produced by virus‐infected cells, and share overlapping biophysical properties, it is important to investigate the specific factors mediating their initial interactions with cells.

Glycosaminoglycans (GAGs) are a major class of glycans that are abundant components of the ECM and basement membranes, where they play key structural and signalling roles (Perez et al. 2023). They have been implicated in the interactions of both HCMV and EVs with cells (Boyle and Compton 1998; Christianson et al. 2013; Compton et al. 1993; Joshi and Zuhorn 2021; Li et al. 2022; Mitra et al. 2021; Perez et al. 2023; Song et al. 2023). GAGs include hyaluronic acid (HA, or hyaluronan), chondroitin sulfate (CS), dermatan sulfate (DS), heparan sulfate (HS), and keratan sulfate (KS) (Perez et al. 2023; Varki 2022). These GAG types differ in their disaccharide composition and sequence, the degree and the pattern of sulfation, and the tissue localization (Varki 2022). Among them, HS and CS are ubiquitous across tissues, found as heparan sulfate proteoglycans (HSPGs) and chondroitin sulfate proteoglycans (CSPGs), respectively (Perez et al. 2023; Varki 2022). Both EVs and HCMV virions have been shown to bind heparin, which resembles the highly sulfated domains of HS, and to utilize HS for binding to various cell types (Boyle and Compton 1998; Christianson et al. 2013; Compton et al. 1993; Joshi and Zuhorn 2021; Li et al. 2022; Mitra et al. 2021; Song et al. 2023).

Despite the initial findings, no studies to date have directly compared interactions of co‐isolated EVs and viruses with GAGs, to understand whether they may utilize the same ligands for cell binding. Such investigation is needed to clarify whether HS is a universal mediator of EV‐cell binding, how viral infection may change these interactions, and whether co‐produced EVs and viruses share overlapping mechanisms of cell ligand engagement. These questions are particularly relevant in the context of the roles played by EVs released by virus‐infected cells, such as the transfer of viral pathogen‐associated molecular patterns (PAMPs) to neighbouring naïve cells (Nolte‐‘t Hoen et al. 2016). Furthermore, identifying the ligands that EVs utilize for cell binding and uptake is crucial for understanding the degree of specificity of EV‐cell interactions, and for the subsequent development of targeted therapeutic EV strategies.

In this work, we sought to characterize and compare the interactions of HCMV and EVs derived from HCMV‐infected or uninfected cells with recipient naive cells, with a focus on the involvement of GAGs in these interactions. Using a GAG oligosaccharide microarray as well as inhibition assays, we first show that both types of EVs interact with heparin. However, in the context of interactions with cells, we find that HS on the cell surface inhibits EV binding to cells, while promoting HCMV binding. The same effect, albeit lower in magnitude, was observed for HS found on the EV surface, indicating that HS represented a bi‐directional negative regulator of EV‐cell interactions in fibroblast cells. In contrast, we identify collagen and fibronectin as non‐GAG ECM components that serve as ligands for EV binding to fibroblast cells.

Collectively, our data shows that HS differentially regulates cell binding by EVs and HCMV, and that EV binding to cells can be inhibited by HS on both the cellular surface as well as the EV surface.

## 2 Materials and Methods

### 2.1 Cell Culture

Human foreskin fibroblast (HFF) cells (clone Hs27; from ATCC) were used in all experiments. The cells were grown in Dulbecco's Modified Eagle's Medium (DMEM; Sigma‐Aldrich) supplemented with 10% fetal bovine serum (FBS), 100 units/mL of penicillin, 100 µg/mL of streptomycin, and 292 µg/mL of L‐glutamine (all from Gibco), in a humidified incubator (Binder) set to 37°C and 5% CO_2_.

### 2.2 Generation of HCMV Stocks, HCMV Infection and Plaque Assay

HCMV strain Merlin R1111, which lacks expression of RL13 and the pentameric complex, was used in all experiments (Stanton et al. 2010). This strain was selected because it yielded large amounts of extracellular viral particles that were needed for cell binding assays. HCMV stocks were generated by infecting confluent HFF monolayers with HCMV at multiplicity of infection (MOI) of 0.01 for 1 h, tilting the flasks every 15 min, and then replacing the diluted virus stock with fresh complete DMEM. Infected HFF cells were then incubated until all cells displayed cytopathic effects and were detaching from the flasks, at 8–10 days. The conditioned medium was collected and clarified by centrifugation at 300xG for 5 min, to remove cells and cellular debris. HCMV stocks were then stored at −80°C. The infectivity of HCMV stocks was determined using a plaque assay. Briefly, 50,000 HFF cells were seeded per well of a 24‐well plate, infected with serial dilutions of HCMV stocks, and overlaid with 1.2% methylcellulose (Sigma‐Aldrich). Three weeks later, plaques were counted and infectivity computed as plaque‐forming units per ml (PFU/mL).

Conditioned medium used for the purification of EVs and viral particles for functional assays was produced by infecting confluent HFFs with HCMV at the MOI of 0.01 (or leaving them uninfected), for 8–10 days, in complete DMEM (supplemented with 10% commercial EV‐depleted FBS; from Gibco). To separate EVs from HCMV, we utilized a method recently reported (Bokun et al. 2025), outlined in Figure 1. Briefly, the conditioned medium was clarified by centrifugation, transferred to centrifuge bottles (35 mL per bottle), underlaid with 5 mL of 16% iodixanol as a density cushion (OptiPrep; Sigma‐Aldrich), and centrifuged at 25,000 × G for 2.5 h. The resulting pellets were collected as purified viral particles, and the supernatant was used for downstream purification and labelling of viral particle‐free EVs.

**FIGURE 1:**
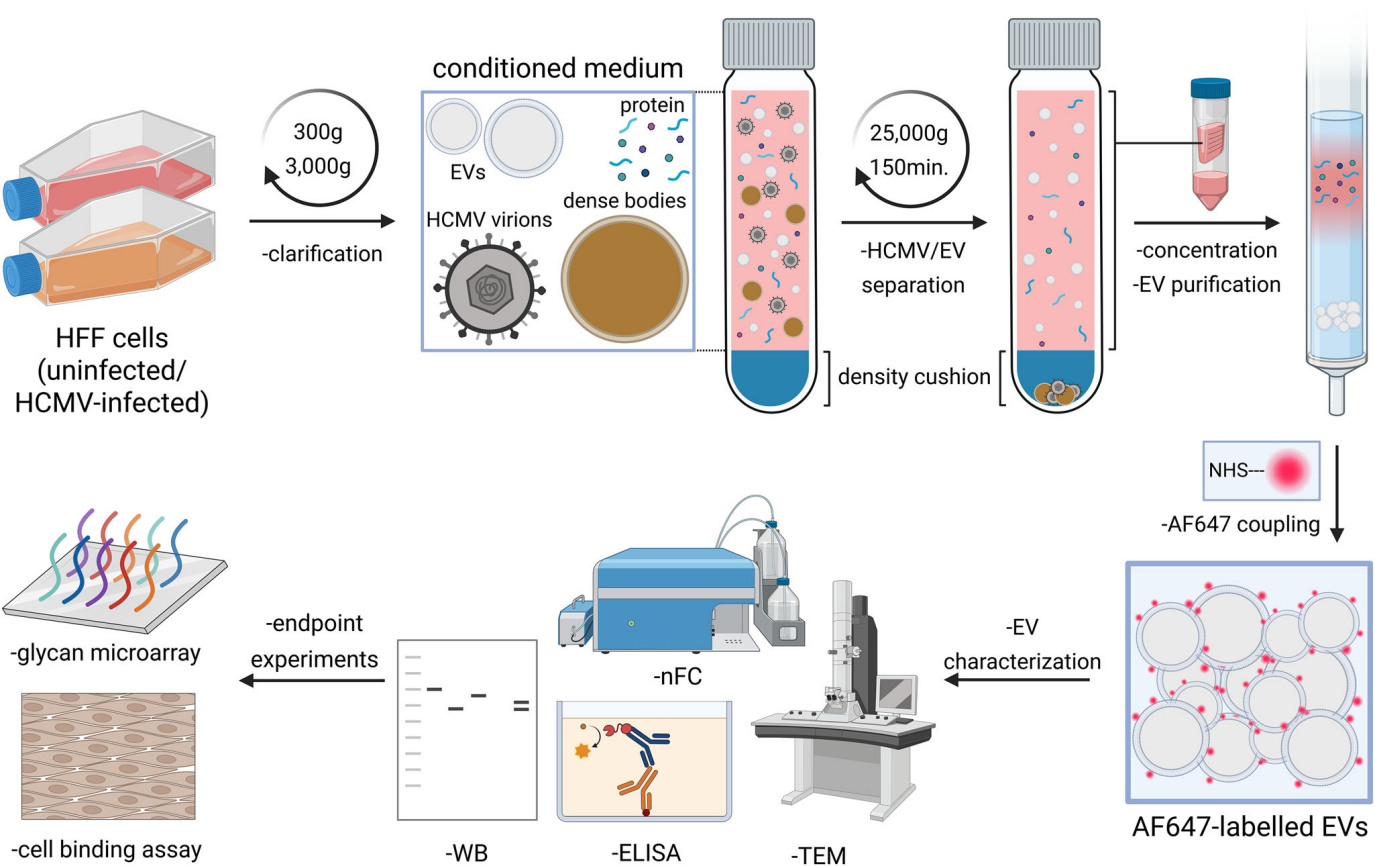
Visual overview of the experimental approach used in the study. HFF cells were grown to confluence and infected with HCMV. Conditioned medium was collected when cells displayed cytopathic effects and clarified by low‐speed centrifugation. The EVs were separated from viral particles using high‐speed centrifugation over density cushions and purified by ultrafiltration and SEC. Purified EVs were coupled with AF647 and characterized using multiple methods before being used in end‐point experiments. WB = western blot; nFC = nano‐flow cytometry; TEM = transmission electron microscopy; NHS = N‐hydroxysuccinimide. Created with BioRender.com.

### 2.3 Purification and Labelling of EVs

Viral particle‐free EVs in 25,000 × G supernatants were concentrated by ultrafiltration on VivaSpin 20 100‐kDa polyethersulfone (PES) columns (Cytiva). The concentrated EV suspension (500 µL) was added to a size exclusion chromatography (SEC) column, made by packing Econo‐Pac 20‐mL columns (BioRad) with Sepharose CL‐2B (Cytiva) to a final bed volume of 20 mL. Dulbecco's Phosphate‐Buffered Saline (DPBS; calcium‐ and magnesium‐free; from Sigma‐Aldrich) was used for washing the column and for elution. Fractions (500 µL) containing EVs, as determined by nano‐flow cytometry (nFC), were pooled and concentrated down to 500 µL using 100‐kDa ultrafiltration (Amicon; from Merck‐Millipore). Concentrated EVs were then labelled with the Alexa Fluor 647 N‐hydroxysuccinimide ester (AF647‐NHS; from Invitrogen) at 100 µg/mL for 1.5 h, at room temperature. The reaction was quenched by addition of Tris‐HCl to 50 mM, and the labelled EVs were re‐purified on the same SEC column, to remove excess labelling reagent/fluorophore. The EVs were re‐concentrated using ultrafiltration, as above, and stored at −80°C. We have submitted all details relevant to EV isolation and characterization to the EV‐TRACK knowledgebase (EV‐TRACK ID: EV250128) (Van Deun et al. 2017).

### 2.4 nFC

All nFC analyses were performed using a flow nanoanalyzer (manufactured by nanoFCM), operated by the accompanying software, NF Profession (v2.12), as described before (Bokun et al. 2025). The instrument was calibrated at each use using quality control (QC) silica nanospheres (from nanoFCM), which were used to optimize laser/filter positioning to maximize signal intensity, and to serve as the concentration standard. Excitation was provided by a 488‐nm laser and side scatter measured through a 488 ± 5 nm bandpass filter. Fluorescence was measured through 525 ± 20 and 670 ± 15 nm bandpass filters, by excitation at 488 and 638 nm, respectively. The sampling pressure was kept constant, at 1.0 kPa. The experimental samples were diluted in DPBS, to obtain 2000–12,000 events per 1‐min acquisition. Both experimental samples and QC nanospheres were acquired for 1 min. Ultrapure water was used as the sheath fluid. The laser power was set to 10 mW and side scatter decay to 2%, except for the QC nanospheres, for which this was set to 20 mW and 0.2%, respectively. The size of experimental particles was determined using silica nanosphere sizing standards (S16Exo; from nanoFCM). A standard curve was constructed from side scatter intensities of the silica nanosphere sizing standards. Both concentration and size were determined in the included software, using built‐in functions. Dot plots and size histograms were also generated in the same software.

CD63 and CD81 on EVs were labelled with recombinant monoclonal antibodies conjugated to FITC and APC dyes (Miltenyi Biotec; items REA1055 and REA513; at 1/100 dilution, in DPBS), respectively, for 30 min at room temperature, and the excess antibody‐fluorophore removed by miniature SEC using ExoSpin columns (Cell Guidance Systems).

### 2.5 Western Blotting for EV‐Associated Proteins

EVs were lysed by addition of 6X RIPA buffer (Pierce) with protease/phosphatase inhibitors (Invitrogen). 6X reducing sample buffer (Alfa Aesar) was added and the mixture heated at 95°C for 5 min. The samples were cooled to room temperature, centrifuged briefly, and loaded onto 3%–8% gradient tris‐acetate 1.0‐mm gels (Invitrogen), along with an Invitrogen high‐molecular weight protein ladder. The gel was run in tris‐acetate running buffer (Invitrogen) at 150 V. Protein was transferred from the gel to PVDF membrane using pre‐assembled transfer stacks on an IBlot 2 semi‐dry transfer system (Invitrogen), using a modified, 10‐min protocol. The membrane was blocked with 3% BSA in DPBS with 0.05% Tween‐20, and probed with primary antibodies at 0.5 µg/mL in blocking buffer, overnight at 4°C. The antibodies were against β‐actin (Sigma‐Aldrich; item A2228), fibronectin (SantaCruz Biotechnology; item sc‐8422), and calnexin (Cell Signalling Technology; item 2433S). The membrane was washed three times with DPBS with 0.05% Tween‐20 and incubated with secondary antibodies conjugated to horseradish peroxidase (HRP) (Dako), at 0.5 µg/mL in blocking buffer, for one hour at room temperature. The membranes were washed twice with DPBS with 0.05% Tween‐20 and once with DPBS. SuperSignal West Pico PLUS chemiluminescent substrate (Invitrogen) was used to detect binding. The membrane was stripped between each probing using the Restore stripping buffer (Thermo Scientific).

### 2.6 Transmission Electron Microscopy

Carbon‐copper support grids (Agar scientific) were first glow discharged for 50 seconds using a modified Fischione NanoClean Model 1070 instrument. Purified EVs were deposited onto the grids for 5 min, blotted off, and then stained with 2% uranyl acetate for 45 seconds. Excess staining solution was blotted off and grids washed with ultrapure water. Grids were then allowed to dry before imaging on an FEI Tecnai T12 Spirit with accelerating voltage of 120 kV, at 6500x and 26000x magnifications.

### 2.7 Enzymatic Digestion of Heparan Sulfate and Chondroitin Sulfate on EVs

EVs were purified from pooled conditioned medium of uninfected cells collected at 7, 8 and 9 days of culture, and labelled with AF647‐NHS as described above. Purified labelled EVs were then either left untreated or treated with either a mixture of heparinase I and III (at 0.6 and 0.3 milliunits/mL, respectively), or chondroitinase ABC (at 30 milliunits/mL) in DPBS (all enzymes were obtained from Sigma‐Aldrich). The digestion was performed overnight at room temperature, with continuous mixing, in the dark. The EVs were then re‐purified on the same Sepharose CL‐2B SEC column used for their initial purification. The generated untreated control and HS‐ and CS‐depleted EVs were characterized by nFC, to measure their concentration, estimate size, and to confirm AF647 labelling. The removal of HS and CS was verified by indirect ELISA.

### 2.8 ELISA for CD63, Heparan Sulfate and Chondroitin Sulfate

The fractions eluting from the Sepharose CL‐2B SEC columns following treatment of EVs with digestion enzymes were analyzed for CD63, HS, and CS. An aliquot (50 µL) of SEC fractions 1–8 was added to wells of a half‐area 96‐well high‐binding plate (Greiner), in duplicate, and allowed to adsorb overnight at room temperature. Fractions were then aspirated from the plate and wells washed with DPBS. The immobilized EVs were blocked with 3% BSA in DPBS, for 1 h at room temperature. Antibodies against CD63 (clone MEM‐259; BioRad item MCA2142), HS (clone 10E4; Amsbio), and CS (clone CS56; Amsbio) were added to the wells, at 1 µg/mL in blocking buffer, for 1 h at room temperature. Wells were then washed three times with DPBS, and HRP‐conjugated anti‐mouse immunoglobulins (Agilent/Dako) added at 0.4 µg/mL in blocking buffer for 1 h at room temperature. The plate was washed three times with DPBS. 3,3',5,5'‐Tetramethylbenzidine (TMB) was added (50 µL per well) until sufficient colour development occurred, which was stopped by addition of 0.5‐M hydrochloric acid (50 µL per well). The plate was read at 450 nm on a Tecan Sunrise microplate reader.

### 2.9 Glycosaminoglycan Microarray Analysis

The generation of the GAG oligosaccharide microarray was recently reported (Tajadura‐Ortega et al. 2025). This included preparation of GAG oligosaccharide probes by conjugating size‐defined oligosaccharide fractions derived from HA, heparin, CS‐A, CS‐B, CS‐C, and KS with a 2‐amino‐N‐(2‐aminoethyl)‐benzamide (AEAB) linker, followed by their immobilization on NHS‐functionalized glass slides. All relevant details of the microarray fabrication, EV microarray binding assays, and the corresponding data analysis are included as supplementary information, in accordance with the MIRAGE (Minimum Information Required for a Glycomics Experiment) guidelines for reporting glycan microarray‐based data (Liu et al. 2017).

### 2.10 Flow Cytometry Analysis of Fibroblast Surface Heparan Sulfate and Chondroitin Sulfate

HFF cells grown in T‐175 flasks were detached from flasks with 10 mM EDTA (at 4°C) and fixed with 4% paraformaldehyde (Pierce) for 5 min. Cells were washed three times by addition of DPBS and centrifugation at 400xG for 10 min. The cells were blocked with 3% BSA in DPBS with 1 mM EDTA for 1 h at room temperature. Antibodies against HS (clone 10E4; Amsbio), or CS (clone CS56; Amsbio) were added at 1 µg/mL in blocking buffer, for 2 h at room temperature. Cells were washed two times with DPBS, and a secondary antibody conjugated to biotin added for 1 h at room temperature (again at 1 µg/mL in blocking buffer). Two washes were performed, and the cells incubated with streptavidin‐AF647, at 4 µg/mL in blocking buffer, for 30 min at room temperature. Cells were washed twice with DPBS, and pellets resuspended in FACS buffer consisting of DPBS with 0.5% BSA and 1 mM EDTA. Cells were analyzed on a FACScalibur (Becton Dickinson) instrument and the histograms showing expression of GAGs generated in the included software, which was used to control the instrument, CellQuest Pro.

### 2.11 Enzymatic Digestion of Heparan Sulfate, Chondroitin Sulfate, Collagen and Fibronectin on HFF Cells

Plated HFF cells were pre‐treated with either a mix of heparinase I and heparinase III (Sigma‐Aldrich), at 1.2 and 0.6 milliunits/mL, respectively, or chondroitinase ABC (Sigma‐Aldrich) at 60 milliunits/mL for 1 h at 37°C. In separate experiments, cells were treated with a mix of collagenase I and dispase II (Gibco) at 0.1 units/mL and 0.6 units/mL, respectively, for 10 min at 37°C. All enzymes were diluted in complete DMEM, and cells incubated with enzyme‐deficient complete DMEM were used as controls.

To verify effective removal of the target GAGs and ECM proteins, HFF cells were stained with anti‐HS (clone 10E4), anti‐CS (clone CS56), anti‐collagen type I (item 14695‐1‐AP; from Proteintech), and anti‐fibronectin (clone EP5; from Santa Cruz Biotechnology) antibodies, all at 1 µg/mL in blocking buffer, for 1 h at room temperature. Cells were washed three times with DPBS and incubated with secondary antibodies conjugated to AF647 or AF488 (Invitrogen) at 1 µg/mL, in blocking buffer, for 1 h at room temperature. Cells were washed three times with DPBS and nuclei stained with DAPI, at 5 µg/mL for 5 min at room temperature. Three DPBS washes were performed, and cells imaged on a Leica SP5 confocal microscope.

### 2.12 EV Cell Binding Assay

AF647‐labelled EVs were adjusted to 5 × 10^10^ EVs/ml in DMEM containing 10% EV‐depleted FBS (Gibco). Fibroblast cells grown on chambered coverslips (Ibidi) were pre‐treated with enzymes as described above and then pre‐chilled for 10 min at 4°C, after which EVs were added for 1 h at 4°C. The cells were washed twice with cold DPBS, fixed with 4% PFA in DPBS for 5 min, and washed three times with DPBS. Nuclear staining was then performed with either 5 µg/mL DAPI (Invitrogen) or 1X SYBR Green (MedChemExpress) for 5 min. Cells were washed twice with DPBS and analyzed for EV binding by confocal microscopy. In addition to the enzyme treatments, in separate experiments EVs were co‐incubated with 20 µg/mL heparin (Sigma‐Aldrich). For experiments involving heparinase I and III and chondroitinase ABC treatments of purified uninfected fibroblast EVs, the EV concentration was adjusted to 2 × 10^10^ EVs/ml and the time of incubation to 1.5 h, due to a lower yield of EVs obtained. During initial optimization of the EV cell binding assay, the cell surface was stained with the Maackia Amurensis Lectin II (MAL II; biotinylated; from Vector labs). Prior to staining, the cells were first blocked with 3% BSA in DPBS. The lectin was diluted to 5 µg/mL in blocking buffer and added to cells for 1 h. This was followed by two DPBS washes, and addition of streptavidin‐PE (Miltenyi Biotec), at 0.5 µg/mL in blocking buffer, for 30 min. All staining steps were performed at room temperature.

### 2.13 Confocal Microscopy for Quantification of Cell Binding by EVs

Fibroblast cells incubated with EVs were imaged on a Leica SP5 confocal microscope, using a 63x oil‐immersion objective (Leica). AF647 conjugated to EVs was excited by a 635‐nm laser at 20% power, and gain adjusted to prevent signal saturation in the collected micrographs. Single focal planes were acquired at Z positions where nuclei area was largest, to ensure consistency. 3 to 5 micrographs were obtained for each condition. Controls consisting of cells incubated with samples processed identically, but without EVs, were captured using identical settings. The format used was 1054 × 1054, with line averaging set to 5 and the scanning speed set to 100 Hz.

Quantification of EV binding was performed in ImageJ. Obtained micrographs of cell‐bound EVs were first subjected to thresholding to remove background noise prior to quantification. This was done uniformly across all micrographs collected in a single experiment. Following this, integrated density of each micrograph was recorded and the number of nuclei in the same micrographs counted manually in ImageJ. Integrated density was then divided by the number of nuclei in the same image, and the resulting values averaged for each condition. The average number of cells in each micrograph was 38, and the resulting average number or cells analyzed per condition was 117. Micrographs showing EV binding in the main figures were adjusted for contrast and brightness, to accentuate the EV signals and facilitate their visualization, while quantification was performed on raw micrographs.

### 2.14 HCMV Cell Binding Assay

HCMV suspension was adjusted to 1 × 10^5^ plaque‐forming units per ml (PFU/mL) (equivalent to approximately 1 × 10^10^ viral particles/mL) and added to confluent fibroblast monolayers pre‐treated with enzymes as described above and pre‐chilled to 4°C, and incubated for 1 h at 4°C. Cells were washed three times with DPBS and frozen at ‐80°C. For quantification of HCMV binding, viral DNA was purified from the frozen cell monolayers for qPCR of viral DNA.

### 2.15 QPCR Quantification of Cell Binding by HCMV

Genomic DNA was isolated from frozen HFF monolayers using the PureLink Genomic DNA Isolation kit (Invitrogen) and quantified by measuring absorbance at 260 nm on a NanoDrop. 20 ng of DNA per reaction was amplified using the BioRad SSO Universal Probes master mix and a commercial TaqMan HCMV primer/probe set (Applied Biosystems, assay ID Vi06439643_s1). 40 cycles of amplification were performed on an ABI QuantStudio 5 qPCR machine (Applied Biosystems) and fluorescence recorded at each cycle. Quantification of fold changes was carried out in the included software based on the ΔΔCt method with GAPDH as the loading control (Applied Biosystems, assay ID Hs02786624_g1).

### 2.16 Solid‐phase EV Binding Assay

To perform a collagen/fibronectin binding assay, biotinylated EVs were first produced using the same protocol outlined in the ‘Purification and labelling of EVs’ section, by substituting NHS‐AF647 with NHS‐PEG4‐Biotin (Invitrogen), at 200 µg/mL.

Collagen type I (from rat tail; from Sigma‐Aldrich) and type IV (from human placenta; from Sigma‐Aldrich) and fibronectin (from human plasma; from Millipore) were adjusted to 5 µg/mL in DPBS and added to half‐area 96‐well high‐binding plates (Greiner), to adsorb overnight at 4°C. The plates were then washed twice with DPBS and blocked with 2% BSA in DPBS (with Ca^2+^/Mg^2+^; from Sigma‐Aldrich), for 1 h at room temperature. EVs were adjusted to 4 × 10^8^ EVs per mL of blocking buffer and added to the plates. The plates were incubated with EVs overnight at 4°C, with gentle shaking. Following the binding stage, the plates were washed 3 times with DPBS, and EVs fixed with 2% paraformaldehyde (Pierce) in DPBS (for 20 min at room temperature). The plates were washed three times with DPBS. Next, streptavidin conjugated with HRP (Invitrogen) was added to the wells for 30 min at room temperature, in blocking buffer. The plates were washed three times with DPBS and TMB (Abcam) added. The colour development was stopped with 0.5‐M hydrochloric acid, and the plates read at 450 nm using a Tecan Sunrise plate reader.

### 2.17 Statistics

A two‐tailed *t*‐test was used in the comparison of the reported EV diameters (from nFC). EV binding intensities were tested for statistical significance by performing paired, two‐tailed ratio *t*‐tests. One‐way ANOVA with Dunnett post‐hoc test was used for the solid‐phase binding assay. Data were deemed statistically significant when *p* < 0.05 (* for *p* < 0.05; ** for *p* < 0.01; *** for *p* < 0.001). All graphs and statistical testing were done in GraphPad Prism v10.2.3.

## 3 Results

### 3.1 EVs Can be Separated From HCMV Viral Particles Based on Density and They Display Characteristic EV Markers

EVs released by HCMV‐infected fibroblast cells and matching uninfected controls were isolated using a previously established method (Bokun et al. 2025), labelled with AF647, and characterized using multiple methods as recommended in ‘Minimal information for studies of extracellular vesicles 2018 (MISEV2018)’ guidelines (Théry et al. 2018) (Figure 1). The purified EVs visualized by TEM displayed the characteristic circular/oval morphology, with no viral particles observed (Figure 2A). Viral particles purified in parallel were, in turn, enriched in HCMV virions, as highlighted in the rightmost micrograph in Figure 2A. Both types of EVs showed similar size profiles by nFC, with a peak at ∼60 nm, and tapering off towards the larger size range (Figure 2B). A small but statistically significant difference in the reported EV diameter of 4.5 nm was noted (*p* = 0.012) (Figure 2C). Using the same technique, we confirmed that the majority of EVs were positive for AF647 (Figure 2D). We also detected both CD63 and CD81, tetraspanins commonly analyzed as surface EV markers (Théry et al. 2018), by nFC (Figure 2E); and beta‐actin, which is incorporated into the EV lumen, by western blotting (Figure 2F). Fibronectin, which can associate with the surfaces of the EVs (Pathan et al. 2019), and calnexin, were also noted in the EV preparations by western blotting (Figure 2F).

**FIGURE 2:**
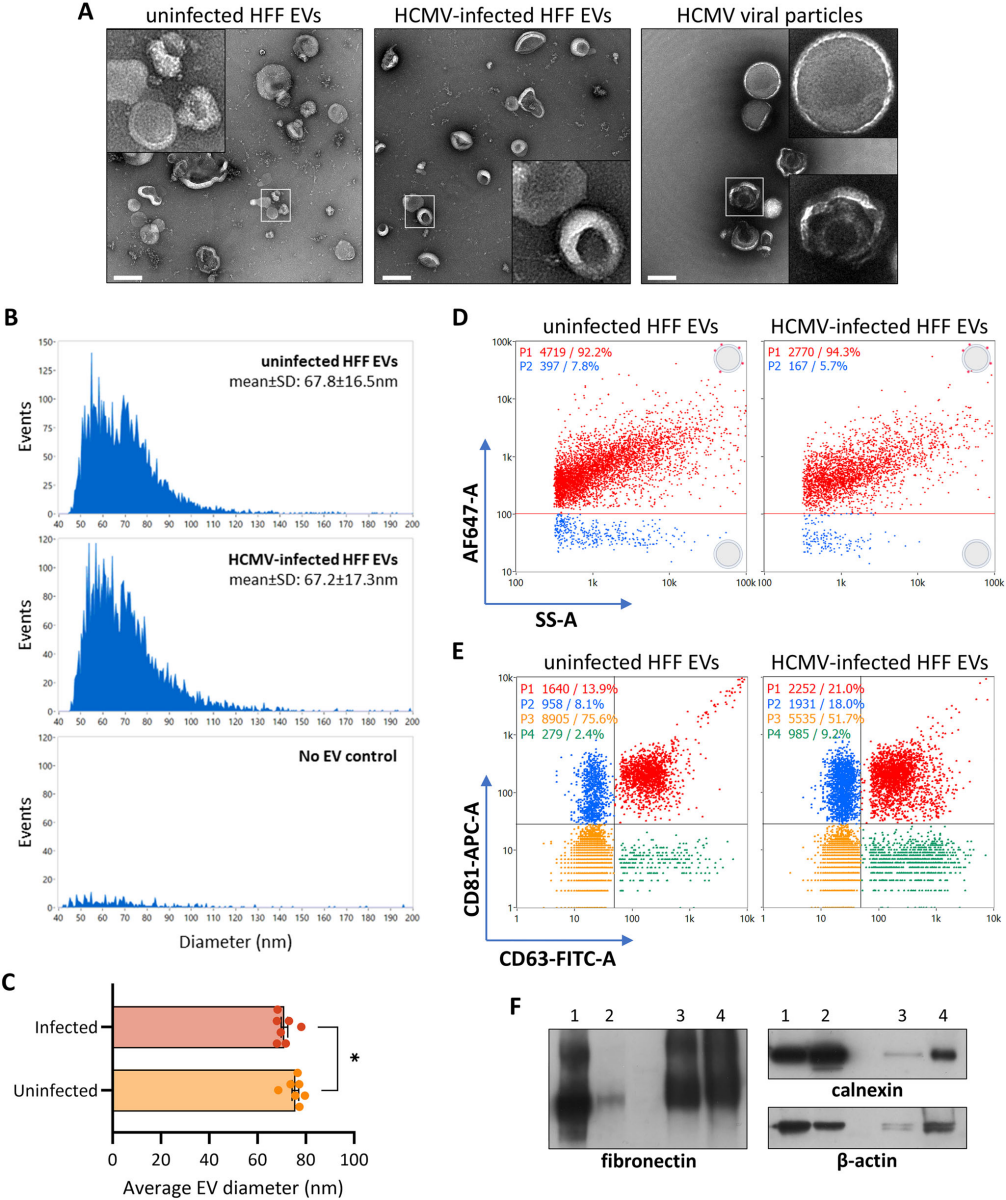
Characterization of end‐purified EVs. Viral particle‐free EVs were purified from uninfected and HCMV‐infected HFF cells as described in Figure 1 and characterized using TEM, nFC, and western blotting. (A) Negative stain TEM revealed EVs with typical oval/circular morphology, and no viral particles in the EV samples purified from HCMV‐infected cells. Viral particles that were purified in parallel are shown in the rightmost micrograph, with the bottom inset showing an HCMV virion and the top inset highlighting a typical subviral dense body. Scale bars = 200 nm. *N* = 3. (B) Representative size distributions of purified AF647‐conjugated EVs produced by uninfected and HCMV‐infected HFF cells, along with a no‐EV control (*N* = 7). Average diameters with SDs are indicated in the top right corners. (C) The average diameter of the EVs from uninfected HFF cells was slightly higher than that of the EVs produced by HCMV‐infected cells (*N* = 7). (D) Both types of EVs were effectively coupled with AF647, as evidenced by nFC (dot plots representative of *N* = 7). (E) The end‐purified EVs were positive for CD63 and CD81 following staining with fluorescent monoclonal antibodies and analysis by nFC (*N* = 1). (E) β‐actin, a protein associated with the EV lumen, was identified in the purified EVs by western blotting. The EVs were also positive for fibronectin and calnexin. *N* = 1. Legend: 1: Uninfected HFF cells; 2: HCMV‐infected HFF cells; 3: Uninfected HFF EVs; 4: HCMV‐infected HFF EVs. A = area; SS = side scatter.

### 3.2 EVs Interact With Heparin and Heparin Inhibits EV‐Cell Interactions

EV binding to GAGs was first assessed using a focused glycan microarray comprising 23 GAG oligosaccharide probes of different types (HA, CS‐A, CS‐B, CS‐C and heparin) with varying chain lengths. EV binding was restricted to heparin, with little to no binding observed for CS and HA (Figure 3A). Binding to heparin was length‐dependent, with longer probes exhibiting higher fluorescence intensities.

**FIGURE 3:**
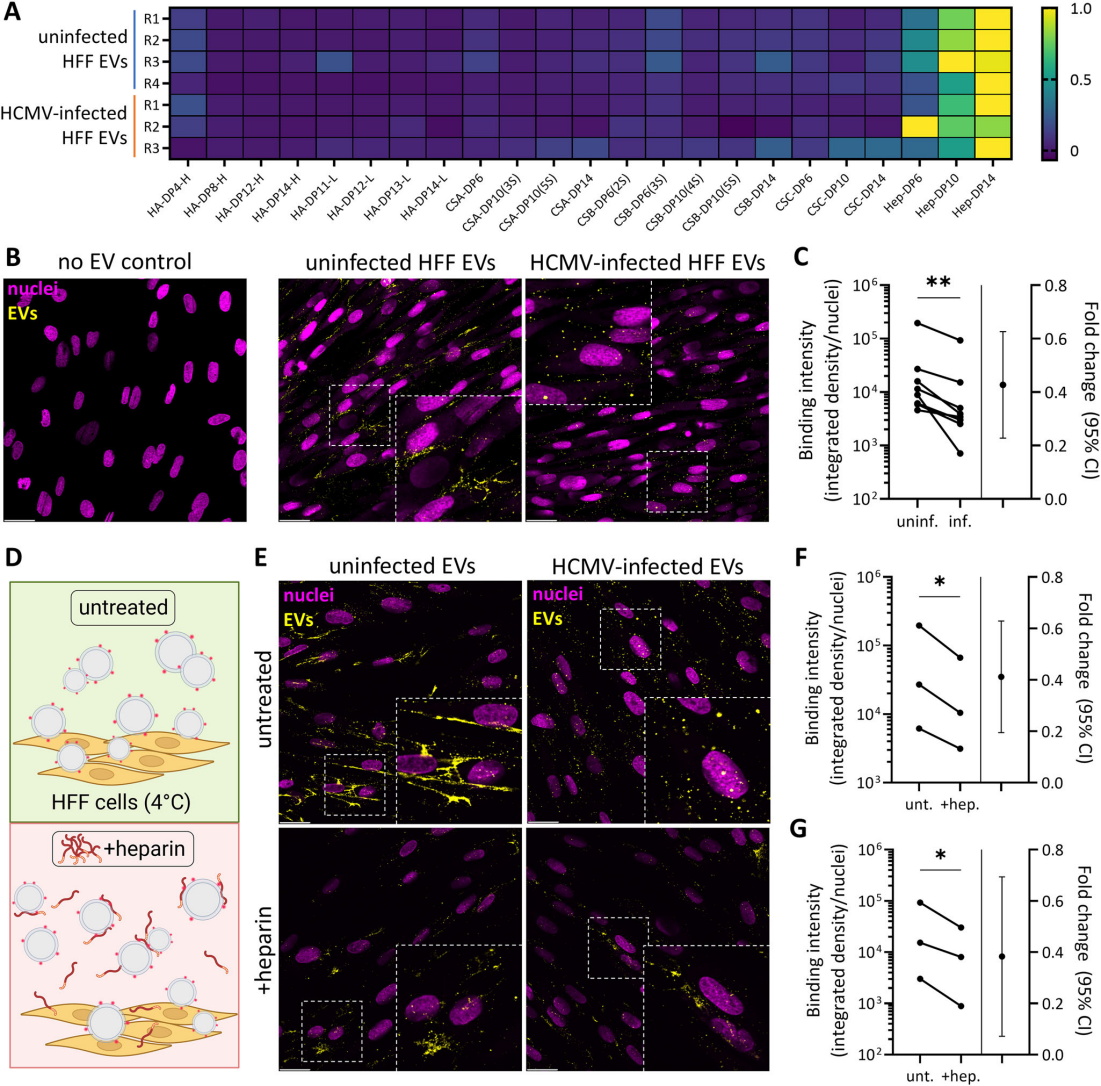
EVs interact with the glycosaminoglycan heparin and heparin inhibits EV‐cell interactions. (A) AF647‐EVs were added to a glycan microarray to profile their glycosaminoglycan binding properties. EVs interacted with heparin in a length‐dependent manner, with little to no interaction with HA and CS (*N* = 4 for uninfected HFF EVs; *N* = 3 for HCMV‐infected HFF EVs). Oligosaccharide probes included in the microarray are indicated below the heatmap. (B) A confocal microscopy‐based EV cell binding assay was set up to investigate the involvement of glycosaminoglycans in EV‐cell interactions. This revealed more uninfected HFF EVs bound to naïve HFF cell surface than the EVs from HCMV‐infected cells, and the former were characterized by a more clustered/continuous binding pattern. (C) EVs from HCMV‐infected HFF cells exhibited lower baseline HFF binding than the EVs from uninfected cells (*N* = 7). (D) To investigate whether heparin inhibited EV binding to cells, exogenous heparin was co‐incubated with EVs at 20 µg/ml. (E). Lower numbers of cell‐bound EVs were observed in presence of exogenous heparin, as evidenced by confocal microscopy. (F, G) Quantification of cell‐bound EV AF647 fluorescence revealed an inhibition of 58.9% and 61.7% for EVs from uninfected and HCMV‐infected HFF cells, respectively, by heparin (*N* = 3). Scale bars in B and E = 30 µm. ‘uninf. = uninfected; ‘inf.’ = infected; ‘unt’ = untreated; ‘+hep’ = +heparin; ‘CI’ = confidence interval. Panel D created with BioRender.com.

To assess whether this translated to cell binding, we established a confocal microscopy‐based EV cell binding assay. Using this approach, AF647‐labelled EVs were detected mostly on the HFF surface, as evidenced by co‐localization with MAL II lectin staining of the α2,3‐linked sialic acids on the cell surfaces (Figure S1) (Geisler and Jarvis 2011). In paired samples, EVs from HCMV‐infected cells displayed a significantly lower baseline cell binding than the EVs from uninfected cells (Figure 3B,C), with a 58% lower signal (*p* = 0.0049). In addition to this quantitative difference, the binding patterns differed; EVs from uninfected cells were found as both discrete events as well as in a continuous/clustered pattern, while the EVs from infected cells were found almost exclusively as discrete events (Figure 3B).

In the presence of exogenous heparin, EV binding to the cell surface was reduced for both groups (Figure 3D,E), with reductions of 58.9% for uninfected HFF EVs (Figure 3F) and 61.7% for HCMV‐infected HFF EVs (Figure 3G) (*p* = 0.017 and 0.031, respectively).

### 3.3 Fibroblast Heparan Sulfate Inhibits Binding by EVs but Promote Binding by HCMV

Having established that EVs from both infected and uninfected cells interact with heparin, we next investigated whether cellular HS contributed to EV binding to fibroblast cells.

We first confirmed the presence of HS and CS on HFF cells by flow cytometry and confocal microscopy (Figure S2). Both GAGs were detected across the cell population by flow cytometry (Figure S2A). Confocal microscopy revealed HS staining as both discrete and continuous signals along the cell surface, while CS staining was largely granular/discrete (Figure S2B). Enzymatic treatments of confluent and sub‐confluent HFF cultures with heparinases I and III or chondroitinase ABC effectively removed HS and CS, respectively, from the cell surface (Figure S2C). Unlike HS and CS, HA and KS were not detected on HFF cells by either method (data not shown).

We next performed EV cell binding assays where removal of HS or CS preceded the EV binding step (Figure 4A). The removal of HS increased binding by uninfected HFF EVs by 2.5‐fold (*p* = 0.0020) and by HCMV‐infected HFF EVs by 1.7‐fold, however the latter was not statistically significant (*p* = 0.176) (Figure 4B–D). In contrast, no changes in binding were observed following CS removal. To compare this with HCMV virions, which are known to utilize HS for attachment (Boyle and Compton 1998; Compton et al. 1993; Mitra et al. 2021), we performed a binding assay in which HCMV binding to HFF cells was quantified by qPCR (Figure 4A). A substantial decrease of 13.2‐fold (or a reduction of 92.4%) was observed upon HS digestion, while digestion of CS had no effect (Figure 4E). These results highlight a differential role for HS in mediating EV/HCMV interactions with HFF cells.

**FIGURE 4:**
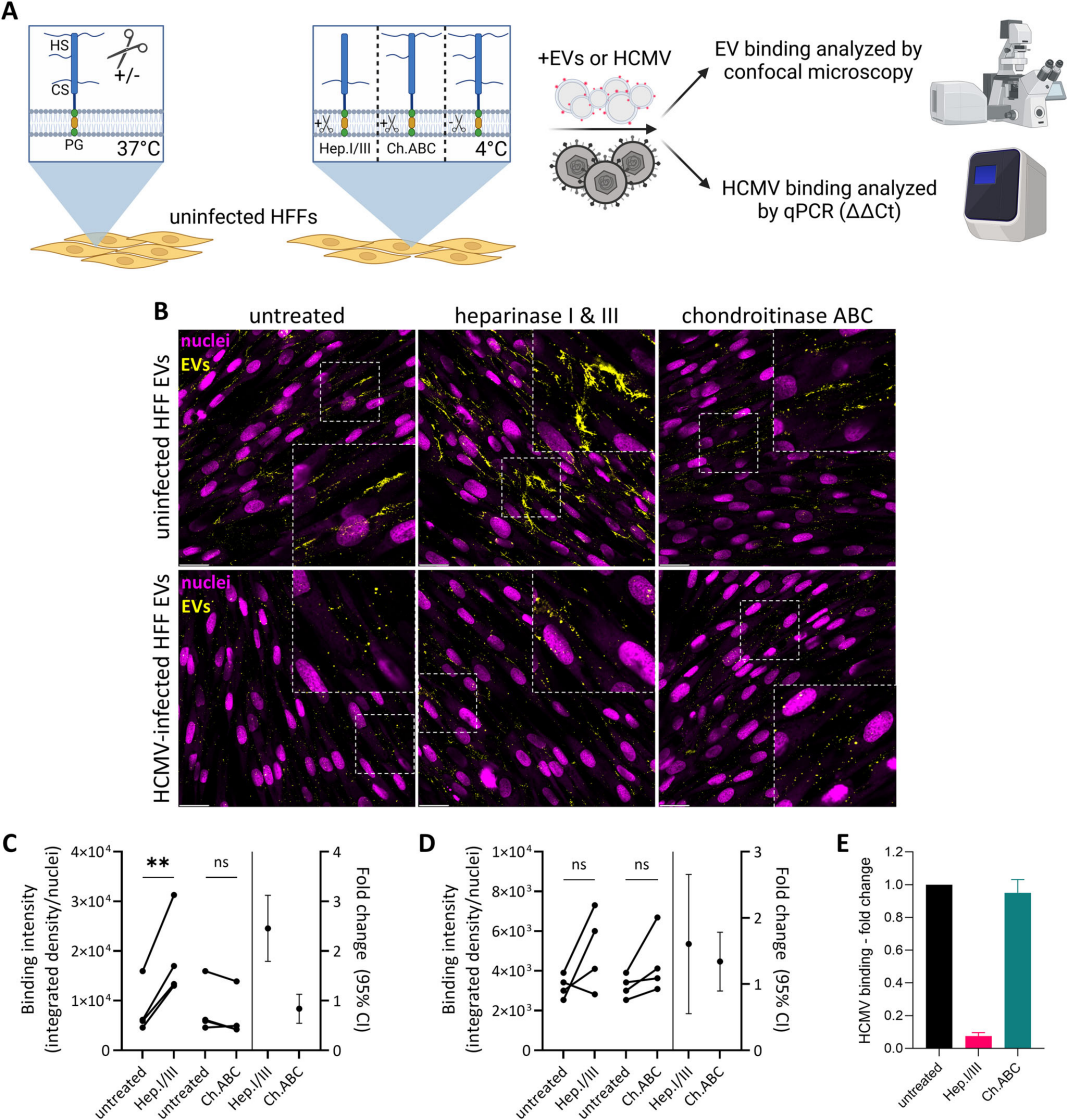
Enzymatic digestion of heparan sulfate on the fibroblast surface promotes EV‐cell interactions. (A) To investigate whether fibroblast heparan sulfate was a binding partner for EVs on fibroblast surfaces, we used a mixture of heparinase I and III, or chondroitinase ABC, to digest HS and CS, respectively. The binding of EVs and HCMV to cells was analyzed by confocal microscopy and qPCR, respectively. (B) Binding of EVs from uninfected HFF cells to naïve HFF cells was increased following heparinase I/III treatment (top), while binding by HCMV‐infected HFF EVs remained largely unchanged (bottom). Binding by both types of EVs was unchanged with chondoritinase ABC treatment (rightmost panels, top and bottom). (C, D) A 2.5‐fold increase in binding was noted following heparinase I and III treatment for EVs produced by uninfected HFF cells (C), while an increase of 1.7‐fold seen with EVs produced by HCMV‐infected cells was not statistically significant (D). (E) HCMV binding to naïve HFF cells was unchanged with chondroitinase ABC treatment and substantially reduced with heparinase I/III treatment of the cells. All data are from 4 independent experiments. Scale bars in B and C = 30 µm. CI = confidence interval. Panel A created with BioRender.com.

### 3.4 EV‐associated Heparan Sulfate Inhibits EV‐Cell Interactions

Having established fibroblast HS as a negative regulator of EV binding, we sought to investigate if HFF EVs themselves are decorated with GAGs and whether these contribute to EV‐cell interactions.

Labelled EVs from uninfected HFF cells were treated with heparinase I and III or chondroitinase ABC (Figure 5A), and their GAG contents quantified by ELISA (Figure 5B). The enzymatic treatments did not appear to shift the EV elution from the SEC column, as evidenced by CD63, a surface marker of EVs (Figure 5B, left). Both HS and CS were identified in the EV‐containing (CD63‐positive) SEC fractions (fractions 1–4), and were specifically removed by their respective enzymes, confirming effective and selective digestion (Figure 5B, middle and right panels). To ensure that enzymatic treatment did not alter EV integrity, induce aggregation, or alter AF647 labelling, we analysed the treated and untreated EVs by nFC (Figure S3). All EV samples showed consistent size distributions and mean particle diameters (Figure S3A), and consistent AF647 fluorescence (Figure S3B), confirming that the digestion did not affect these parameters.

**FIGURE 5:**
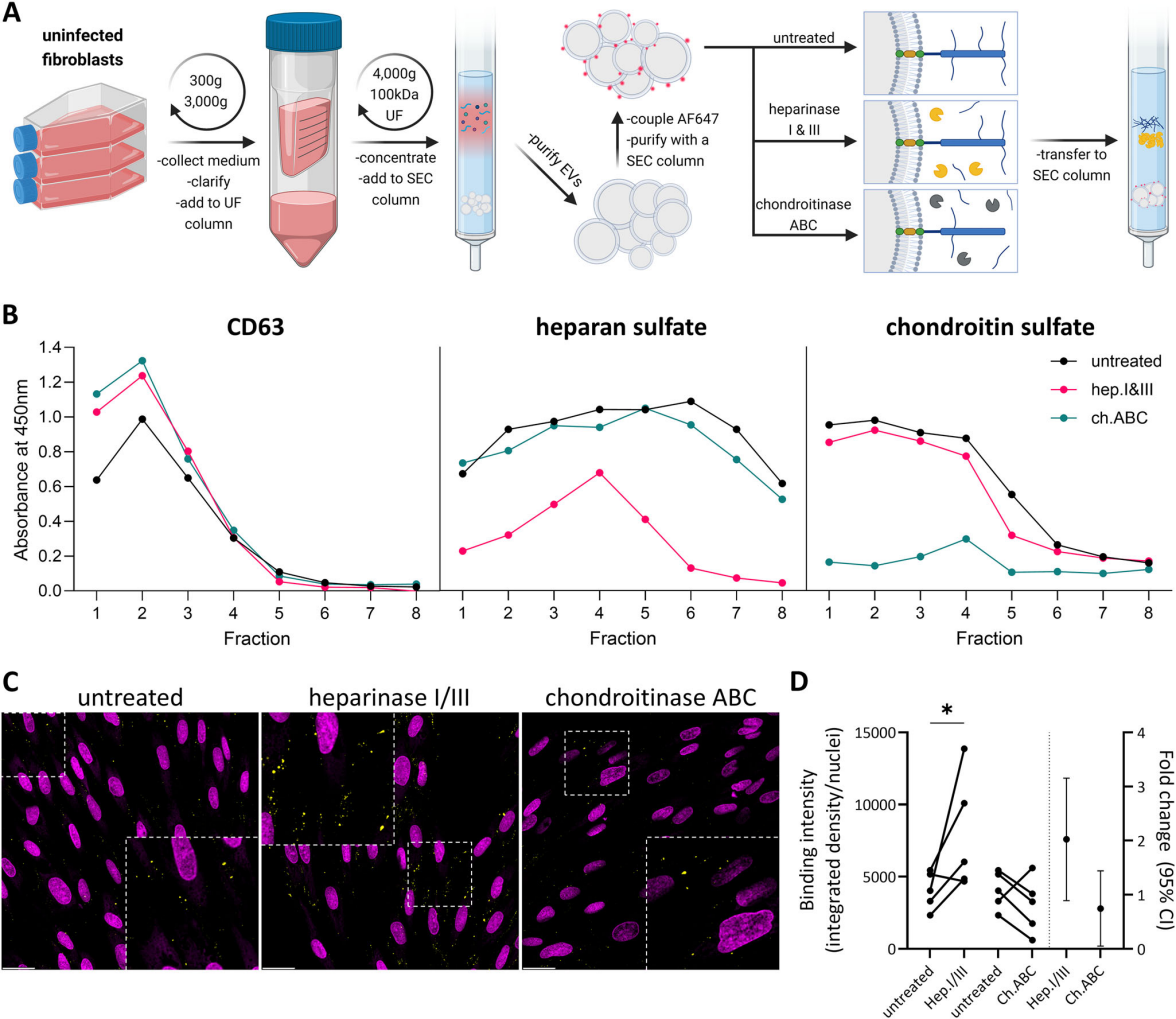
Heparan sulfate is displayed on the EV surface and inhibits EV binding to cells. (A) To investigate whether glycosaminoglycans are found on the EV surface and if they play a role in interactions with cells, EVs were purified from clarified conditioned medium by ultrafiltration and SEC and labelled with AF647 as described previously. The EVs were split across three equal parts and treated with either a mixture of heparinases I and III, or chondroitinase ABC, or left untreated. The digestion products and enzymes were removed by re‐purifying EVs by SEC. (B) CD63, an EV surface‐associated protein, HS and CS were evaluated in each fraction by ELISA. CD63 was found in those fractions expected to contain EVs (fractions 1–4), and the positivity was unchanged with enzyme treatment. HS and CS contents were specifically reduced with the enzymatic treatments employed. (C) An equal number of EVs were added to HFF cells to evaluate cell binding by confocal microscopy. EVs treated with heparinases I and III displayed increased cell binding compared to chondroitinase ABC‐treated and untreated EVs. (D) A 2.0‐fold increase in cell binding was noted with EVs treated with heparinases I and III compared to untreated EVs (*N* = 5). No major changes were observed for the chondroitinase ABC‐treated EVs. Scale bars in C = 30 µm. CI = confidence interval. Panel A created with BioRender.com.

When the HS‐ and CS‐depleted EVs were analyzed for cell binding by confocal microscopy (Figure 5C), HS‐depleted EVs displayed a 2‐fold higher binding than untreated EVs (*p* = 0.044), while binding by CS‐depleted EVs was unchanged (Figure 5D). This demonstrated that HS on EVs, in addition to HS on cells, played a role in inhibiting EV‐cell interactions, while EV CS did not play a role in this process.

### 3.5 Collagen and Fibronectin Promote Cellular Surface Binding by EVs

Having demonstrated that HS is not a relevant binding target for EVs on fibroblasts, we next investigated whether other major ECM components—specifically collagen and fibronectin—mediated these interactions.

Using a solid‐phase binding assay, we found that EVs bound to collagen type I and fibronectin, but not collagen type IV (Figure 6A,B). To investigate whether these proteins represented genuine binding partners on fibroblast cells, we confirmed their presence on HFF cells by immunostaining (Figure S5, upper row), and, as with HS and CS, verified effective removal using a mixture of collagenase and dispase enzymes (Figure S4). Confocal microscopy analysis of EV binding (Figure 5C,D) showed that the removal of collagen/fibronectin caused a reduction of 37.7% for uninfected EVs (*p* = 0.017) (Figure 6E) and 51.3% for HCMV‐infected EVs (*p* = 0.0038) (Figure 6F), confirming that these ECM components indeed contributed to EV interactions with fibroblast cells.

**FIGURE 6:**
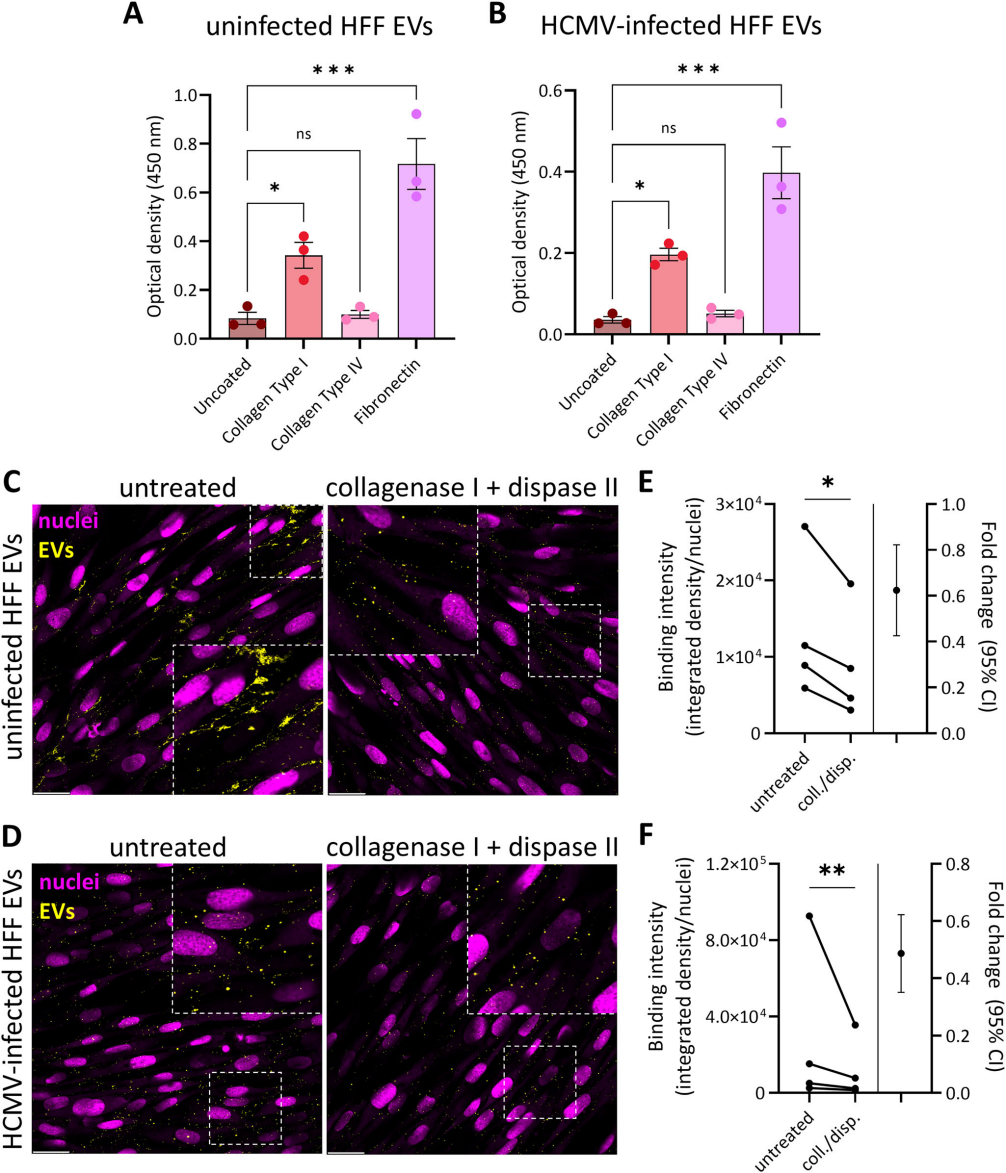
Collagen type I and fibronectin mediate EV binding to the fibroblast surface. (A, B) To investigate which non‐glycosaminoglycan ECM factors may serve as binding partners for EVs, a solid‐phase binding assay was performed. This revealed that EVs produced by both uninfected (A) and HCMV‐infected HFF cells (B) interacted with collagen type I and fibronectin, but not collagen type IV (one‐way ANOVA; *N* = 3). (C, D) When collagen and fibronectin were digested from the HFF surfaces using a combination of collagenase I and dispase II, lower numbers of EVs were observed bound to the HFF surfaces. (E, F) Quantification of EV binding revealed reductions in binding of 37.7% for the uninfected (E) and 51.3% for the HCMV‐infected HFF EVs (F) (*N* = 4). Scale bars in C and D = 30 µm. CI = confidence interval.

## 4 Discussion

Understanding the mechanisms that mediate interactions of EVs with cells is critical to elucidating how EVs traffic between cells, the specificity of their uptake, and how this may affect the development of future EV‐based therapeutics. In this study, we investigated the role of GAGs in modulating these interactions, focusing on EVs derived from both uninfected fibroblast cells and those infected with HCMV. We report, for the first time, that HS can inhibit EV‐cell interactions in a bi‐directional manner, while promoting HCMV attachment. These findings highlight HS as a differential regulator of EV and HCMV binding to fibroblast cells.

In the first part of this work, we confirmed that fibroblast EVs interacted with heparin, as shown before for EVs produced by multiple different cell types (Atai et al. 2013; Balaj et al. 2015; Barnes et al. 2022; Christianson et al. 2013; Reiter et al. 2019; Zhou et al. 2023). However, in the context of fibroblast interactions, cellular HS did not serve as a relevant binding partner for EV binding, which stood in contrast with previous reports (Atai et al. 2013; Balaj et al. 2015; Barnes et al. 2022; Christianson et al. 2013; Cerezo‐Magaña et al. 2021; Reiter et al. 2019; Zhou et al. 2023). These seemingly counterintuitive results showing binding of EVs to heparin and the lack of dependence on cellular HS for cell binding can be explained by several factors. Firstly, heparin is more highly sulfated, and therefore more negatively charged than HS, a feature that enables it to interact with a wider range of binding partners, often with higher affinity (Varki 2022). Given this, it is likely that EV interactions with HS are of insufficient affinity to support stable surface binding. In line with this, digestion of HS from fibroblast surfaces could expose higher‐affinity binding partners, thereby enhancing EV binding. HS does contain heparin‐like domains, however the availability and the spatial organization of these domains in the fibroblast ECM may not be conducive to productive EV binding. Heparin has also been reported to aggregate EVs in solution (Atai et al. 2013), a phenomenon that could inhibit cell binding by EVs in a way unrelated to interactions with surface HS. Thus, the observed inhibition by heparin likely does not inherently and automatically reflect a functional role for HS as a cell binding partner for EVs.

Despite HS not serving as a binding target for EVs on fibroblast cells, heparin binding represents a relevant property exploitable for purification of EVs. This has already been described for EVs derived from multiple cell types (Balaj et al. 2015; Barnes et al. 2022; Reiter et al. 2019; Zhou et al. 2023), and our data adds to the growing evidence that binding to heparin may be a common feature amongst EVs produced by all cell types, and as such a potential candidate for widely applicable EV purification or concentration. However, one previous report identified two distinct sub‐population of EVs, one which interacted with heparin and another one that did not (Zhou et al. 2023), highlighting a potential bias if heparin is implemented for EV isolation. This would also be in line with our data, wherein heparin did not completely inhibit EV binding to fibroblast cells, indicating that there was a fraction of EVs that did not interact with heparin. It is also worth noting that the observed binding to heparin on the glycan microarray required a higher gain setting than routinely employed in our group, and that the resulting signals were lower than most other samples analyzed (Tajadura‐Ortega et al. 2025), suggesting that EV binding to heparin is either of lower affinity, or that there is only a subpopulation of EVs that bound to heparin. Despite the growing literature showing EV binding to heparin, the identities of heparin‐interacting molecules on EVs have not been confirmed, however it is known that heparin‐binding proteins such as annexins, collagens, fibronectin, vitronectin and others are found or even enriched in EVs (Buzás et al. 2018; Wang et al. 2025). Studies employing genetic and/or biochemical approaches to disrupt these components may reveal which of them mediate binding to heparin and other GAGs.

As revealed by our experiments, yet another factor that contributes to the inhibition of EV binding to cells is the presence of HS on the EV surface. Our study shows that HS on cell surfaces, but also EV surfaces, can play an inhibitory role in EV‐cell interactions in a bi‐directional manner. As mentioned, HS, like heparin, is negatively charged, so it is possible that HS existing on both the cell surface and the EV surface causes electrostatic repulsion of EVs that is reduced upon removal of HS from either the cells or the EVs. This is in line with previous reports of EVs carrying a negative charge (Midekessa et al. 2020; Wang et al. 2025), which could be reduced by removal of charged glycans (Williams et al. 2019), and a report of electrostatic interactions playing a role in milk EV adsorption to lipid bilayers (Ridolfi et al. 2023). While an early study of the potential role of EV‐associated GAGs in EV‐cell interactions did not reveal any changes in these interactions upon removal of GAGs on EVs from the U‐87 MG glioblastoma cell line (Christianson et al. 2013), a recent preprint employing a genetic approach reported a greatly enhanced uptake of HEK293F EVs when GAGs were not present on either the EV membrane or the recipient cell membrane, consistent with observations made here (Tian et al. 2026). The importance of HS and other GAGs on EVs extends beyond just regulating interactions with cells, as GAGs are known to interact with and sequester growth factors, cytokines, and other signalling factors (Varki 2022).

Another novel aspect of our work is that we examined EVs released from HCMV‐infected cells and, in some experiments, the HCMV virus itself, to make multi‐directional comparisons. This enabled us to conclude that co‐produced HCMV and EVs displayed contrasting properties, wherein HCMV relied on HS as its dominant attachment factor, whereas EVs were inhibited by HS. Additionally, while we did not detect major differences in the two types of EVs when it comes to HS inhibition, we did confirm that the EVs produced by infected cells had reduced baseline cell binding compared to those from uninfected cells. This suggests that the EV factors that enable interactions with cells are dysregulated during HCMV infection, and that the trafficking of these EVs is different than in homeostatic conditions.

In contrast to findings concerning HS, this study highlights collagen and fibronectin as genuine binding partners for both uninfected and HCMV‐infected HFF EVs on the fibroblast surface. Both proteins were previously demonstrated to be bound by EVs produced by B cells (Clayton et al. 2004), while fibronectin has been identified as a component of the EVs that can mediate interactions with cells, including via HS and integrins (Chanda et al. 2019; Elsharkasy et al. 2025; Osawa et al. 2017; Purushothaman et al. 2016). However, the role of fibronectin on the cellular surface in this process has not been investigated previously. Several types of EVs were shown to interact with collagen type I in hydrogels (Tam et al. 2024) and in pull‐down experiments (Palmulli et al. 2023), as well as with laminin (Arif et al. 2024; Isogai et al. 2025). These reports, including ours, demonstrate the complex and multi‐layered nature of the EV‐cell interactions, and highlight the need for further, systematic investigation in this area. Likewise, while not directly investigated in our work, the GAG contents of the EVs themselves may differ in infection and homeostatic conditions, arising from changes in glycosylation pathways induced by viral infection, thus contributing yet another dimension to the complexity of the interactions between the EVs and recipient cells. Related to this, it has become evident that EVs possess a secondarily acquired proteome in form of an EV ‘corona’, which, while not directly addressed in this work, adds to the complexity of EV‐cell interactions (Buzas 2022).

Our findings are restricted to fibroblast cells as both EV‐ and HCMV‐producing cells and recipient cells, comprising a model for interactions *in cis*. It is important to highlight that both the cellular and the EV surface proteome differ in different cell types, entailing different potential interaction partners/pathways. Therefore, it is possible that the reason for the opposite conclusions made previously was that the experiments were carried out in other cell types, including cancer cells. Therefore, further investigation is required to ascertain how variable the involvement of HS in EV‐cell interactions is across different cell types. Another limitation of this work, but also most previous EV research in general, is the heterogeneity of the EVs (Bordanaba‐Florit et al. 2021). Because commonly utilized methods like SEC isolate EVs in bulk, without separation of individual subtypes, it is impossible to delineate the GAG‐ and cell‐binding properties of individual EV subtypes (e.g. exosomes and microvesicles). Therefore, as discussed previously, it is possible that there are subpopulations of EVs in our samples with distinct heparin/HS‐binding properties, as also reported previously (Zhou et al. 2023).

This study represents the first application of a glycan microarray to biochemically profile the interactions between EVs and glycans, with a narrow focus on GAGs. However, future efforts should be expanded to other classes of glycans that may mediate interactions between cells and EVs, including N‐glycans and O‐glycans, and glycolipids (Varki 2022). These glycans can terminate with sialic acid and/or fucose residues, which could mediate interactions with EVs, as shown for multiple viruses (Stencel‐Baerenwald et al. 2014). Glycan microarrays have been a powerful screening tool that have enabled assignments of numerous protein‐glycan and pathogen‐glycan interactions (Heimburg‐Molinaro et al. 2024; Li and Feizi 2018), and they could similarly be used to better understand which glycans may mediate EV‐cell interactions.

This study contributes novel insights into the role of HS in regulating EV‐cell interactions, into how EVs and viruses may differ in this regard, and should stimulate novel studies, either targeted or wide‐ranging, concerning these interactions.

## Author Contributions

**Vladimir Bokun**: conceptualization, investigation, writing – original draft, writing – review and editing, visualization, methodology, formal analysis, data curation, funding acquisition. **Antonio Di Maio**: methodology, validation, resources, visualization. **Yibing Zhang**: methodology. **Wengang Chai**: methodology, resources, conceptualization, writing – review and editing. **Beth Holder**: conceptualization, funding acquisition, writing – review and editing, project administration, supervision, resources. **Yan Liu**: supervision, resources, project administration, writing – review and editing, funding acquisition, conceptualization.

## Funding

The glycan microarray studies were supported by the Wellcome Trust Biomedical Resource Grants (WT099197/Z/12/Z, 108430/Z/15/Z and 218304/Z/19/Z) and the March of Dimes Prematurity Research Centre grant [22‐FY18‐82]. V.B. was supported by the Imperial College London President's PhD scholarship. V.B. and B.H. were also supported by funding from the National Institute of Allergy and Infectious Diseases (National Institutes of Health; award R01AI185558). Additional funding was from the Genesis Research Trust, to V.B., Y.L., and B.H. Acquisition of the nanoFCM flow nanoanalyzer was funded by a Wellcome Trust Multi‐User Equipment grant (UNS108435; to BH). The content is solely the responsibility of the authors and does not necessarily represent the official views of the National Institutes of Health.

## Conflicts of Interest

The authors declare that they have no conflicts of interest with the contents of this article.

## Supporting information




**Supporting Information**: jev270390‐sup‐0001‐FigureS1‐S4.pptx

**Supporting Information**: jev270390‐sup‐0002‐SuppMat.xlsx

**Supporting Information**: jev270390‐sup‐0003‐SuppMat.doc

**Supporting Information**: jev270390‐sup‐0004‐SuppMat.xlsx

**Supporting Information**: jev270390‐sup‐0005‐SuppMat.docx

## Data Availability

The data that support the findings of this study are available from the corresponding author upon reasonable request
