# Supplementary material for: Heparan Sulfate Differentially Regulates Fibroblast Binding by Extracellular Vesicles and Human Cytomegalovirus
Source: J Extracell Vesicles. 2026 Nov 3;15(11):e70390. doi: 10.1002/jev2.70390 (PMC13730358; doi:10.1002/jev2.70390)
Supplement: Supplementary file 1 [file JEV2-15-e70390-s003.docx]

**Supplementary** **Glycan Microarray Information Template**

**Based on MIRAGE Guidelines (1)**

|  | | | **Description*** |
| --- | --- | --- | --- |
| 1. **Sample: Glycan Binding Sample** | | | |
| Description of Sample | Extracellular vesicles (EVs) were purified from uninfected human foreskin fibroblast cells (HFFs), or those infected with human cytomegalovirus (HCMV), at multiplicity of infection of 0.01, as described previously (2). The conditioned medium containing EVs and HCMV was collected when all cells displayed cytopathic effects and were detaching from the growth vessels. The collected medium was clarified by low-speed centrifugation, and the EVs separated from HCMV viral particles by centrifugation at 25,000xG for 2.5 hours over density cushions, at 4°C. The EVs remaining in the supernatant following high-speed centrifugation were recovered, concentrated by ultrafiltration (100 kDa), and purified from medium contaminants using a Sepharose CL-2B size exclusion chromatography column. Purified EVs were then concentrated using ultrafiltration and saved at -80°C. | | |
| Sample modifications | The EVs were conjugated with Alexa Fluor 647 (AF647), for visualisation of binding to the glycan microarray and cells. This was done using an AF647 NHS ester (Invitrogen), by incubating it with the EVs for 1.5 hours at room temperature. The excess reagent was quenched with Tris and EVs re-purified using the Sepharose CL-2B column. The labelling was validated by nano-flow cytometry. | | |
| Assay protocol | EVs conjugated to AF647 were diluted in DPBS to 5x10^10^ particles/ml and added to the microarray slide for 1.5 hours at room temperature, then washed three times with DPBS, followed by fixation with 4% paraformaldehyde for 20 minutes at 4°C. The microarray slide was then washed three times with DPBS, and once with ultrapure water, before drying. | | |
| **2.** **Glycan Library** | | | |
| Glycan description for defined glycans | The microarray of 48 probes (in-house designation “Sequence Defined Array Set 1”) contains a collection of sequence-defined glycosaminoglycans (GAGs) with different chain lengths and sulfation patterns, including hyaluronic acid, chondroitin sulfate A, B, and C, heparin, and keratan sulfate. The full list of probes is shown in Figure 3 (panel A) and is also available, together with their names and structures, as supporting information. | | |
| Glycan modifications | The glycans were functionalized with 2-amino-N-(2-aminoethyl)-benzamide (AEAB) linkers, which allowed printing onto NHS-functionalized glass slides. | | |
| 1. **3.** **Printing Surface; e.g., Microarray Slide** | | | |
| Description of surface | Nexterion™ 3-D Hydrogel (H) glass microarray slide with NHS-ester functionalized hydro-polymer coating. | | |
| Manufacturer | SCHOTT | | |
| Custom preparation of surface | Not relevant | | |
| Non-covalent Immobilization | Not relevant | | |
| **4. Arrayer (Printer)** | | | |
| Description of Arrayer | Nano-Plotter 2.1 (GeSim, Radeberg, Germany) | | |
| Dispensing mechanism | Non-contact liquid delivery with four dispensing tips. | | |
| Glycan deposition | Each probe was printed in quadruplicate, at approximately 0.7 nL per spot. | | |
| Printing conditions | Glycan probes in phosphate buffer (pH 8.0) were added to a 384-well microtiter plate for printing. The printing processes were conducted under ambient conditions, with relative humidity maintained between 55% and 60%. The printing time was kept within 8 hours. | | |
| 2. **5.** **Glycan Microarray with “Map”** | | | |
| Array layout | | Each array slide contained 16 pads (blocks). Each pad was set up for printing up to 69 probes, in quadruplicate (four spots per probe and four probes in a row). | |
| Glycan identification and quality control | | The quality control work has been carried out previously (3). | |
| 3. **6. Detector and Data Processing** | | | |
| Scanning hardware | Genepix 4300A (Molecular Devices, Berkshire, UK) | | |
| Scanner settings | The slides were scanned using a 635-nm laser and the standard red filter (661 nm to 690 nm; 670DF40). The laser power was set to 100% and gain was changed from 400-800 to identify the optimal setting. A gain setting of 600 was used for quantification. Pixel resolution was set to 5 µm and line averaging was set to 1. | | |
| Image analysis software | Genepix Pro 7 (Molecular Devices, Berkshire, UK) | | |
| Data processing | Fluorescence intensities of microarray spots, corrected for local background, were determined in the included software, Genepix Pro 7. Means of four technical replicates were calculated and blank-corrected using a buffer (DPBS) control. 3 or 4 biological replicates were included for EVs from uninfected and HCMV-infected HFF cells, respectively. The binding intensities, expressed as fluorescence intensities, were normalized to probes yielding the highest signal (1.0) and fractions of maximal binding calculated therefrom. | | |
| **7.** **Glycan Microarray Data Presentation** | | | |
| Data presentation | Data were presented as a heatmap showing all biological replicates analyzed, in Figure 3A.  Only binding to HA, CS, and heparin probes was shown in the main manuscript.  Complete raw data are available as supporting information. | | |
| 4. **8.** **Interpretation and** **Conclusion from Microarray Data** | | | |
| Data interpretation | No software or algorithms were used to interpret processed data. | | |
| Conclusions | EVs produced by both uninfected and HCMV-infected HFF cells interacted exclusively with heparin, while little to no interaction observed with chondroitin sulfate and hyaluronic acid. | | |

*Only items relevant to microarray experiments need to be included in this document which may be cited in manuscripts as a Supplementary Table. Descriptions and references cited here should complement rather than duplicate the content of the Materials and Methods.

**References**

1.	Liu Y, McBride R, Stoll M, Palma AS, Silva L, Agravat S, et al. The minimum information required for a glycomics experiment (MIRAGE) project: improving the standards for reporting glycan microarray-based data. Glycobiology. 2017;27(4):280–4.

2.	Bokun V, Strang BL, Pantazi P, Liu Y, Holder B. Nano-Flow Cytometry-Guided Discrimination and Separation of Human Cytomegalovirus Virions and Extracellular Vesicles. Journal of Extracellular Vesicles. 2025;14(5):e70060.

3.	Tajadura-Ortega V, Chai W, Roberts LA, Zhang Y, Di Maio A, Decout AC, et al. Identification and characterisation of vaginal bacteria-glycan interactions implicated in reproductive tract health and pregnancy outcomes. Nat Commun. 2025;16(1):5207.
