# Supplementary material for: Heparan Sulfate Differentially Regulates Fibroblast Binding by Extracellular Vesicles and Human Cytomegalovirus
Source: J Extracell Vesicles. 2026 Nov 3;15(11):e70390. doi: 10.1002/jev2.70390 (PMC13730358; doi:10.1002/jev2.70390)
Supplement: Supplementary file 2 [file JEV2-15-e70390-s004.doc]

| **Requirement** | **Please Include Requested Information** |
| --- | --- |
| 1.1. Purpose | We utilized nano-flow cytometry for the determination of concentration, size, and Alexa Fluor 647 conjugation in extracellular vesicles (EVs) derived from uninfected and human cytomegalovirus (HCMV)-infected human foreskin fibroblast cells (HFFs). The aim of the study was to compare glycosaminoglycan binding properties of the two types of extracellular vesicles and the human cytomegalovirus viral particles, as well as their fibroblast binding properties. |
| 1.2. Keywords | cytomegalovirus; extracellular vesicles; glycosaminoglycans; glycans; extracellular matrix. |
| 1.3. Experiment variables | Extracellular particles secreted by uninfected and HCMV-infected HFFs in the conditioned medium were separated by high-speed centrifugation, yielding viral particle-free EVs and EV-free viral particles. In addition, EVs were also purified using the same protocol from uninfected control cells. |
| 1.4. Organization name and address | Organization: Institute of Reproductive and Developmental Biology (IRDB), Department of Metabolism, Digestion and Reproduction, Faculty of Medicine, Imperial College London  Address: Du Cane Road, London W12 0NN, United Kingdom |
| 1.5. Primary contact name and email address | Vladimir Bokun - v.bokun20@imperial.ac.uk |
| 1.6. Date or time period of experiment | 2020-2025 |
| 1.7. Conclusions | NA |
| 1.8. Quality control measures | The instrument is adjusted and calibrated with each use using quality control nanospheres (from nanoFCM). Blanks are run in parallel with each use and the counts recorded routinely do not exceed 200 events per 1-min acquisition. Side scatter measurements are calibrated using silica nanospheres of known diameters (from nanoFCM). |
| 2.1.1.1. (2.1.2.1., 2.1.3.1.) Sample description | EVs secreted by HFF cells (uninfected and HCMV-infected), purified from conditioned medium by high-speed centrifugation, size exclusion chromatography, and ultrafiltration, stored aliquoted at -80°C. |
| 2.1.1.2. Biological sample source description | Human foreskin fibroblasts (HFFs) were obtained from ATCC and grown in DMEM supplemented with exosome-depleted fetal bovine serum (10%) and penicillin and streptomycin.  HCMV strain Merlin R1111 was obtained from Dr Richard Stanton’s laboratory at University of Cardiff School of Medicine. This HCMV strain was derived from clinical material and captured in a bacterial artificial chromosome (BAC) before being transfected into permissive cells for reconstitution of mature infectious virus stocks. |
| 2.1.1.3. Biological sample source organism description | NA |
| 2.1.2.2. Environmental sample location | NA |
| 2.3. Sample treatment description | Viral particles used in the study were not treated. EVs used in the study were conjugated with Alexa Fluor 647 using N-hydroxy succinimide ester (NHS ester), followed by re-purification by size exclusion chromatography and ultrafiltration. |
| 2.4. Fluorescence reagent(s) description | N-hydroxy succinimide ester (NHS) ester (from Invitrogen) was resuspended in DMSO at 10 mg/ml as a 100X stock. This reagent couples Alexa Fluor 647 covalently via primary amine groups of amino acids containing them. Separately, antibodies against CD63 and CD81 conjugated to FITC and APC, respectively, were used to stain the EVs. The details of the antibodies/reagents and the staining protocol are included in the methods section of the manuscript. |
| 3.1. Instrument manufacturer | nanoFCM |
| 3.2. Instrument model | Flow nanoanalyzer |
| 3.3. Instrument configuration and settings | The instrument is equipped with 488-nm and 638-nm lasers and 488/10, 525/40, 580/40, 670/30 and 710/40 filters. Side scatter and fluorescence signals are detected by 3 single photon counting modules (SPCMs). In our experiments, we measured side scatter by excitation at 488 nm at 10 mW through the 488/10 filter, and Alexa Fluor 647 (or APC) fluorescence by excitation at 638 nm (at 10 mW) through the 670/30 filter. FITC was detected through the 525/40 filter with excitation at 488 nm at 10 mW. Sampling pressure was kept constant at 1.0 kPa and experimental samples were analyzed under the same conditions as quality control nanospheres that were used for concentration measurements. The instrument was adjusted at each use using the quality control nanospheres, and blanks were run each time. EV sizing was determined based on side scatter intensities of silica nanospheres of different sizes as standards (from nanoFCM). The running of samples, the analysis, and the creation of dot plots shown in the manuscript were done in the NF Profession software, v2.13. |
| 4.1. List-mode data files | We did not provide the data files associated with the study as the file output from the flow nanoanalyzer instrument is of .nfa format and therefore cannot be opened in standard flow cytometry analysis software. FCS files can be made available upon request from the authors. |
| 4.2. Compensation description | No compensation was required. |
| 4.3. Data transformation details | NA |
| 4.4.1. Gate description | Linear gates dividing the Alexa Fluor 647-positive and -negative population of EVs were drawn to assess positivity. Also, quadrant gates were drawn to assess CD63 and CD81 positivity of EVs. |
| 4.4.2. Gate statistics | Percentages of EVs that were positive/negative are displayed in dot plots. |
| 4.4.3. Gate boundaries | Gates are shown in dot plots as necessary; no gate coordinates are included. |
